# An Everyday Patient-Centered Discussion Model for Primary Care: Protocol for a Feasibility and Acceptability Study of the Zeroing in on Individualized, Patient-Centered Decisions (ZIP) Approach

**DOI:** 10.2196/64998

**Published:** 2025-10-08

**Authors:** Sarah Skurla Dorin, Frances B Schulenberg, Stephanie Visnic, Bradley Youles, Rob Holleman, Jeremy B Sussman, Tanner J Caverly

**Affiliations:** 1 Center for Clinical Management Research VA Ann Arbor Healthcare System Ann Arbor, MI United States; 2 Institute for Research on Innovation & Science Survey Research Center Institute for Social Research University of Michigan Ann Arbor, MI United States; 3 Department of Internal Medicine University of Michigan Medical School Ann Arbor, MI United States; 4 Department of Learning Health Sciences University of Michigan School of Medicine Ann Arbor, MI United States

**Keywords:** shared decision-making, lung cancer screening, blood pressure treatment, primary care, discussion model

## Abstract

**Background:**

The Zeroing in on Individualized, Patient-Centered Decisions (ZIP) approach was developed to be a feasible, everyday shared decision-making (SDM) approach to personalizing decisions in primary care. Current SDM models, which require 5 to 10 minutes just to present initial information, are impractical in primary care, highlighting the need for more concise, patient-centered approaches. The ZIP approach preserves core aspects of SDM while offering a more pragmatic framework suited to real-world clinical constraints. This approach includes three key elements: (1) making a personalized recommendation, (2) qualitatively presenting trade-offs, and (3) supporting patient decisional autonomy. Previous work has found this approach to be acceptable. However, little is known about how feasible and acceptable the ZIP approach is during an actual primary care visit.

**Objective:**

This paper aims to describe the protocol for a pilot test of the feasibility and acceptability to both patients and primary care physicians (PCPs) of using a paper-based deployment of the ZIP approach in a primary care clinic.

**Methods:**

Two case studies were examined: lung cancer screening (LCS) and blood pressure (BP) treatment decisions. This study was a multicomponent pilot implementation study involving training PCPs in the ZIP approach and providing them with an encounter-based decision aid supporting the ZIP approach during clinic visits. Eligible patients were either candidates for an initial LCS conversation or a conversation about intensifying BP medication. The patient-PCP medical encounters were audio recorded. Following the appointment, the patient completed a short survey and underwent a semistructured interview. After PCPs completed 2 to 3 study appointments, they underwent a semistructured interview reflecting on their experience with the ZIP approach. Surveys and interviews sought to understand the overall ZIP components presented during the appointment (ie, feasibility) and the extent to which patients and physicians found the approach appropriate (ie, acceptability). Survey data were analyzed to provide an overview of patient and physician demographics. Interviews were transcribed and analyzed through qualitative coding and thematic analysis to identify high-level takeaways of the feasibility and acceptability of this approach.

**Results:**

This study was funded in October 2022 by the Department of Veterans Affairs. We recruited 10 PCPs and 23 patients (n=4, 17% patients undergoing LCS and n=19, 83% patients involved in BP treatment decision-making). Data collection took place from October 2023 to April 2024. Data analysis concluded in December 2024. Planned paper submission will occur in June 2025.

**Conclusions:**

The results from this pilot implementation study will contribute to the ongoing efforts toward integrating a practical approach to SDM into primary care. This pilot will lay the groundwork for an effective and efficient larger-scale trial.

**International Registered Report Identifier (IRRID):**

DERR1-10.2196/64998

## Introduction

### Background

During a routine clinic visit, primary care physicians (PCPs) often have only 1 to 2 minutes to discuss a single preventive service [1,2]. While using shared decision-making (SDM) to discuss these preventive care options is often promoted in guidelines, current models of detailed SDM typically require up to 15 to 20 minutes for each decision [1,2]. This creates a clear incongruity between the time available for SDM in primary care and the time needed by current models. To address this gap and make SDM more feasible for primary care, our research team previously developed the everyday SDM approach [1]. This approach was designed to take only 2 to 3 minutes to complete while still focusing on core elements of SDM such as collaborative communication. This makes it a potentially more feasible and practical approach for implementation in the fast-paced primary care setting compared to traditional detailed SDM approaches.

Although initially called “everyday SDM,” the approach was renamed to Zeroing in on Individualized, Patient-Centered Decisions (ZIP) based on early feedback from PCPs. The name change was intended to counter widespread physician concerns and perceptions that SDM is not feasible within the constraints of primary care settings [3]. Previous research has shown the ZIP approach’s theoretical acceptability through focus groups with patients [2]. Specifically, patients and PCPs who engaged in feedback sessions regarding the ZIP approach felt that the 3-step process was an acceptable compromise approach to SDM: “it provided the patient with a more personalized recommendation” and supported the patient receiving extra information compared to traditional encounters [2]. The ZIP encounter focuses on three key elements: (1) making a personalized recommendation, (2) briefly presenting qualitative information on key trade-offs, and (3) conveying full support for decisional autonomy and desires for more information on the part of the patient.

### Objectives

While the concept of ZIP was appealing to patients and a potentially more feasible SDM approach for physicians, it remains unknown how ZIP can be integrated into primary care appointments. Specifically, there are questions about whether it realistically can be completed in 1 to 2 minutes and the extent to which patients and PCPs approves of this approach when carried out in actual clinical practice. To address these gaps, we present the protocol for a single-arm, nonrandomized pilot study testing the feasibility and acceptability to both patients and PCPs of using a paper-based deployment of the ZIP approach in a primary care clinic. The aim of this study was to evaluate the real-world acceptability and feasibility of the ZIP approach during actual primary care visits, focusing on 2 everyday primary care decisions in which a brief, effective, patient-centered approach is necessary—lung cancer screening (LCS) and blood pressure (BP) management.

## Methods

### Overview

This study was a multicomponent pilot implementation study involving physicians and their patients. The pilot focused on personalizing two decisions through ZIP: (1) how strongly to encourage starting annual low-dose computed tomography LCS for patients meeting current US Preventive Services Task Force eligibility criteria and (2) how strongly to encourage intensification of BP treatment (ie, prescribing a BP medication for the first time, adding another BP medication to the current regimen, or increasing the dose of current medications) for primary prevention of atherosclerotic cardiovascular disease. Importantly, discouraging LCS or BP treatment for low-benefit or ineligible patients was not part of this pilot. Pilot implementation included a physician education session on the ZIP approach, an audio-recorded medical encounter between the physicians and study patients, a postappointment survey and interview with the patient, and a postintervention interview with the physician.

### Ethical Considerations

The VA Ann Arbor Healthcare System institutional review board (IRB) initially approved this study on February 9, 2023 (1725106). Any protocol amendments were submitted for IRB approval, and all relevant updates were shared with the study team and documented internally.

Study team members obtained informed consent from all individuals before activities involving human participants. The research team complied with informed consent guidelines and adhered to local, national, and international law and regulations regarding the protection of personal information, privacy, and human rights. Original IRB approval and participant consent cover secondary analysis without additional consent.

All data were deidentified to safeguard participant information. For confidentiality, every eligible patient was assigned a unique patient ID number. The study principal investigator and research team analysts had access to the deidentified dataset.

Patient participants were remunerated with a US $25 Meijer gift card for their time.

### Paper-Based Decision Aid Development

The basis for the intervention was to present the physician with tailored guidance based on the intervention’s individualized net benefit for the patient—and have the PCP engage in the ZIP approach based on this tailored information. To accomplish this, we created a paper-based decision aid to personalize decisions for LCS and BP treatment based on well-validated prediction models and a previously developed web-based tool [4-11]. We generated a personalized paper-based decision aid for each study patient that was presented to the PCPs before the appointment and provided personalized patient information to support the use of ZIP. The decision aid included each patient’s relevant risk information (ie, pack years, quit status, and chronic obstructive pulmonary disease or emphysema diagnosis for LCS and high-density lipoprotein and total cholesterol, systolic BP, smoking status, and diabetes for BP treatment). Risk inputs were obtained from the patients’ medical records and used to calculate each patient’s individualized net benefit for LCS or BP treatment. The tool provided information on how preference sensitive the decision was for the patient (ie, the degree to which net benefit depended on how the person weighed the pros and cons), as well as other personalized risk information (eg, the number needed to screen to avoid 1 death).

**Figure 1:**
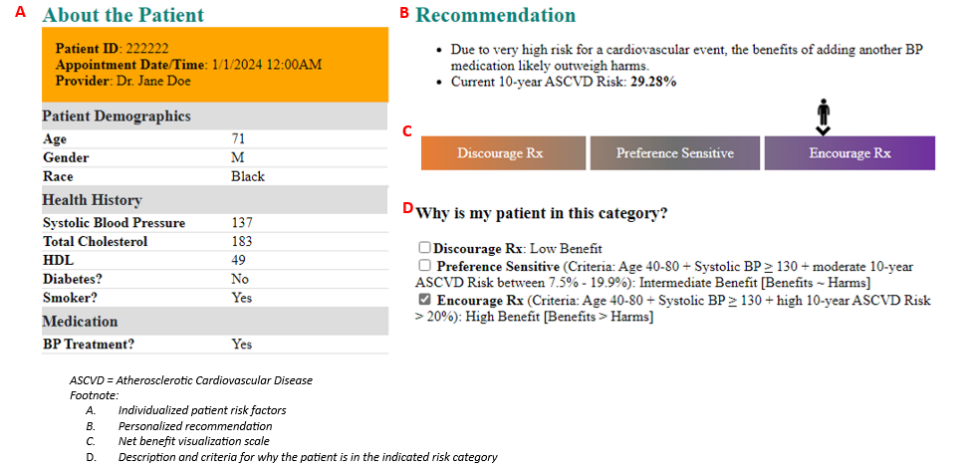
Blood pressure decision aid and net benefit visualization scale example.

On the basis of each person’s calculated individualized net benefit, patients were placed along a net benefit spectrum that included a preference-sensitive zone and an encouragement zone (Figure 1). The preference-sensitive zone meant that the patient was eligible for care but their risks and benefits were in fine balance, so neither strong encouragement nor discouragement was warranted, and conditional guidance was best. The encouragement zone, on the other hand, communicated that the net benefit was more clear-cut for the patient due to a high chance of net benefit (ie, the benefits of receiving the intervention likely outweighed the harm). In this case, PCPs were guided to more strongly encourage screening or BP treatment. Table 1 presents the sample 30-second personalized recommendation scripts for patients in the encouragement zone versus the preference-sensitive zone. On the basis of early PCP feedback, scripts were edited and restructured into bullet point format for easier delivery and use during the appointment. Both script versions are shown in Table 1. The recommendation scripts were discussed during the ZIP training and provided to PCPs within the paper-based decision aid during the pilot. These scripts outlined all aspects of the ZIP approach, were tailored based on the recommendation zone (preference sensitive or encouragement), and served as a starting point for dialogue. The personalized recommendation was what the PCP initially shared—and this could include stronger encouragement in cases in which net benefit was very high (eg, smoking cessation). Regardless of the strength of the initial recommendation, the rest of the ZIP conversation incorporated patient input and preferences and emphasized the patient’s right to make a final decision.

On the basis of previous work and recommendations in national guidelines [12], LCS patients were included in the preference-sensitive zone if they had <16.2 days of life gained and in the encouragement zone if they had ≥16.2 days of life gained from screening [12,13]. For personalizing decisions about whether to intensify a patient’s BP treatment, we categorized patients in the preference-sensitive zone if they had a 10-year atherosclerotic cardiovascular disease risk between 7.5% and 19.9% and in the encouragement zone if they had a risk of ≥20% based on risk thresholds discussed in national guidelines [14-16]. Examples of the full personalized decision aids and further details on how they were created can be found in Figure 1 and Multimedia Appendix 1.

**Table 1:** Zeroing in on the Individualized, Patient-Centered Decisions approach recommendation scripts.

		Version 1: narrative	Version 2: bulleted
**LCS ^a^**			
	**Preference sensitive**		
		“I wanted to talk to you about lung cancer screening. It’s something to consider, but for you it’s not a clear decision. There’s some pros and cons. The big pro is a chance of catching the cancer early and curing it, but there are cons like a risk of false positives, little dots on the CT scan that end up not turning into cancer, leading to more follow-up CTs, with the potential of more risks later on. The good news is you’re not at super-high risk.... You know it’s a little bit of benefit with a little bit of downside and it’s really a personal choice between how you feel about those pros and cons. What are your thoughts?”	“I wanted to talk to you about lung cancer screening. It’s something to consider; for you it’s not a clear decision. There are some pros and cons. The big pro is a chance of catching the cancer early and curing it.” “But there are also cons like a risk of false positives which are little dots on the CT scan that end up not turning into cancer. This could lead to follow-up CTs.” “The good news is you’re not at super-high risk; it’s a little bit of benefit with a little bit of downside. So, it’s really a personal choice between how you feel about those pros and cons.” “What are your thoughts?”
	**Encouragement**		
		“I wanted to talk to you about lung cancer screening. Due to your high lung cancer risk, I think it’s a good idea for you, the benefits are fairly high, you can catch the cancer early, but there are some downsides in that we can find false positives, little dots on the CT scan that end up not turning into cancer. This could lead to follow-up CTs, with the potential of more risks later. But in your case, I think it is worth it because you’re still in pretty good health, your risk of lung cancer is pretty high, and so overall, I’d recommend it. What are your thoughts about that?”	“I wanted to talk to you about lung cancer screening. Due to your high lung cancer risk, I think it’s a good idea for you.” “The benefits are fairly high, you can catch the cancer early. There are also some downsides in that we can find false positives, little dots on the CT scan that end up not turning into cancer. This could lead to follow-up CTs.” “But in your case, I think it is worth it because you’re still in pretty good health and your risk of lung cancer is pretty high. So overall, I’d recommend it.” “What are your thoughts about that?”
**BP^b^ treatment decisions**			
	**Preference** **sensitive**		
		“I’d like to talk to you about whether or not we should start an additional blood pressure medicine. I think this is an individual decision that many people may want, and many people may not want. The good news is you have already gotten your blood pressure to where your risk of heart attacks, strokes, and kidney disease is pretty low for your age. But it would be a bit lower with an additional medicine. If you are the type of person that doesn’t mind taking another medicine if it will lower your risk of heart attacks, kidney disease, and strokes even a little, I think it is a good thing for you. If you are the type of person that only wants to take another medicine if it is a lot of benefit, I think it would be very reasonable to not and to continue to evaluate and monitor you. What are your thoughts?”	“I’d like to talk to you about whether or not we should intensify your blood pressure medicine.” “I think this is a personal decision that many people may want to do, and many people may not want to do.” “The good news is you have already gotten your blood pressure to where your risk of heart attacks, strokes, and kidney disease is pretty low for your age.” “But it would be a bit lower with an additional medicine [or a higher dose of your current medicine].” “So, if you are the type of person that doesn’t mind taking another medicine if it will lower your risk of heart attacks, kidney disease, and strokes even a little, I think it is a good thing for you.” “If you are the type of person that only wants to take another medicine if it has a lot of benefit, I think it would be very reasonable to continue to monitor your blood pressure instead.” “What are your thoughts?”
	**Encouragement**		
		“I’m going to recommend an additional medication. The reason is because your risk of heart attacks and strokes has gone up. It is normal for your risk to go up with age. But we have found that by adding this additional medication it decreases your blood pressure more over the course of the day. What your blood pressure is during today in the office is not important, it is what it is when you are around and active. We have found that by decreasing that we can protect your brain, your kidneys, and your heart. I don’t push medicines, but we have great evidence that as we get older our risk for these things increases, and we can decrease this so you are low risk for your age. It’s just one additional medication, if you have any side effects, which are rare, we can stop it. So I strongly recommend us trying that. What are your thoughts?”	“I’m going to recommend we intensify your BP medication. Your risk of heart attacks and strokes has gone up; it is normal for your risk to go up with age.” “But we have found that additional medication can help because it decreases your blood pressure over the course of the whole day. This helps protect your brain, kidneys, and heart.” “What your blood pressure reading is in the office today is not what’s most important. What’s important is your blood pressure when you are around and active over the course of the day.” “It’s one additional medication [or a higher dose of a medication]. If you have any side effects, which are rare, we can stop it. So, I strongly recommend us trying that.” “What are your thoughts?”

^a^LCS: lung cancer screening.

^b^BP: blood pressure.

### Physician Recruitment and Education

We recruited 10 PCPs with at least 50% of their time devoted to ambulatory care from a single US Department of Veterans Affairs (VA) medical center. Purposeful sampling was conducted to ensure representation of sex and a variety of ages. Eligible PCPs were initially asked to participate in the study through email. The recruitment email described the study and included the consent form. If there was no response after initial contact, follow-ups were made through a second email and then Microsoft Teams messaging. Up to 4 contact attempts were made. Once the PCP agreed to participate and electronically signed the consent form, a team member scheduled them for an initial education session.

The initial education session was a 1-hour meeting in which the physicians first watched a 25-minute video created by the study team highlighting the ZIP technique, specific gaps it fills within primary care and preventive care, and how it can specifically be leveraged for LCS and BP treatment decision patients. The 3 steps of ZIP and how they can be completed were highlighted in the video, providing audio clips and example scripts for going through these 3 steps when initiating a ZIP conversation. The video then concluded with an overview of the study and its various aspects. Following the conclusion of the video, the PCPs were given an opportunity to ask questions and discuss the ZIP approach or the study components with the study team. Once all questions were sufficiently answered, and if time remained, PCPs participated in a patient scenario activity where they could practice the ZIP approach with hypothetical patients. These scenarios were also emailed to all participating PCPs before seeing their study patients.

### Patient Recruitment

We sought to recruit 2 to 3 patients from each PCP, with a target of at least 1 LCS and 1 BP treatment decision patient. Eligible patients were selected if they had an upcoming primary care appointment and were considered eligible for a conversation on starting LCS or intensifying BP treatment. Patients with missing data were excluded as calculating patient risk and benefit level was not possible with missing inputs.

Specific criteria for LCS and BP treatment decision exclusion were determined based on model input requirements. Eligibility for the LCS cohort was based on the US Preventive Services Task Force criteria, which included patients aged 50 to 80 years with a smoking history of at least 20 pack years. Patients were excluded if they had quit smoking over 15 years before, received LCS in the past, undergone a chest computed tomography in the previous year, or been previously diagnosed with lung cancer. These exclusion criteria helped identify any low-benefit patients. Evaluating smoking status and previous tests and diagnoses helps identify any research participants who will derive limited benefit from screening and focus on those who will derive a high benefit instead.

Eligibility for the BP cohort included patients aged 45 to 80 years with a systolic BP between 130 and 160 mm Hg. These criteria ensured a population that was potentially eligible for our study and had a reasonably elevated BP. Patients were excluded for the following reasons: if, in the previous 5 years, they had had a documented history of myocardial infarction or heart disease, loop diuretic use, dementia, or metastatic cancer and if they had 0 measures in the previous 5 years for high-density lipoprotein and total cholesterol or BP or if their race was unlisted. These exclusion criteria aimed to exclude participants already on BP medication for reasons other than primary prevention. Regarding factors such as dementia and metastatic cancer, our team considered any life expectancy considerations to be more important than BP intensification.

Eligible patients were identified through medical chart review. Every 2 weeks, the research team extracted a cohort of eligible patients with a primary care appointment in the following 2 weeks from the VA Corporate Data Warehouse. For confidentiality, every eligible patient was assigned a unique patient ID number. These patients were mailed a recruitment letter informing them of the study and inviting them to participate, along with a copy of the consent form. A study team member then called each patient 2 to 3 days after the study materials were mailed to assess their interest in participating. Up to 3 attempts were made to contact each patient via phone call. Patients who agreed to participate were called 1 day before their appointment as a reminder. Patients were enrolled in the study between October 31, 2024, and April 24, 2025.

### Intervention

PCPs were contacted through Microsoft Teams 1 to 2 days before the appointment to inform them of the upcoming appointment with a study patient and review a copy of the patient-specific decision aid. Patients were asked to arrive 30 minutes before their scheduled appointment, and a study team member met them in the waiting room. The patient’s willingness to participate was confirmed, the components of the study were reviewed (ie, audio-recorded medical encounter and postencounter survey and interview), and the patient’s signed informed consent was obtained. Participants were informed that they could contact the study team through provided contact information with any questions or concerns. The patient was given a digital voice recorder (DVR) and instructions on its use. Patients were encouraged to practice turning on and off the device and shown how to pause the DVR if they did not want a portion of their appointment to be recorded. Patients were informed that the recording would be used to understand the communication style and language that their physicians used during the appointment. Patients were guided to turn on the DVR at the beginning of their appointment. Finally, the patient was handed the paper-based personalized decision aid, which they were instructed to give to the nurse at the beginning of the appointment to then be given to their PCP. Due to the low risk of patient participation, there were no prespecified criteria for modifying the intervention. In addition, there were no concomitant care restrictions during the study.

During the appointment, PCPs carried out the ZIP discussion as instructed based on the information in the patient-specific decision aid.

After the appointment, the study team member met the patient in the waiting room and brought them back to a private room within the primary care unit to administer the postencounter survey and interview. The DVR was returned, and the patient was given a copy of their signed consent form for their records. Patients were administered a survey that asked about demographics, general health information, and satisfaction with and general preferences regarding health care (see Multimedia Appendix 2 for the full survey). Questions focused on assessing overall medical trust, the patient-physician relationship, and BP– or lung cancer–specific decision-making during the appointment. After completing the survey, patients participated in a 10- to 20-minute interview focusing on the conversation with their PCP. Specifically, the interview asked about how the conversation went, the strength of the PCP’s recommendation, and the patient’s final decision regarding screening or medication (see Multimedia Appendix 3 for the full interview guide). LCS patients were asked to compare their perceptions of the benefits and harms of undergoing a screening test and share how worried they were about lung cancer and how helpful they thought screening would be at mitigating this concern. BP treatment decision patients were asked to compare the benefits and harms of BP medication and share how worried they were about myocardial infarction and stroke and how helpful BP medication was in reducing this risk. These interviews were audio recorded. After completing the survey and interview, each patient received a US $25 gift card as remuneration for their time.

### Physician Interview

After PCPs completed study visits with 2 to 3 patients, they were contacted to schedule a 30-minute interview and a short demographic survey (see Multimedia Appendices 4 and 5 for the full interview guide and survey, respectively). During these semistructured interviews, the PCPs were asked to reflect on using the ZIP approach in primary care. The PCPs were also asked to provide feedback on the sample 30-second discussion script, specifically on the script’s language and how strictly they stuck to the script during the intervention. Physicians were asked how receptive their patients were to this intervention and whether they planned to continue using this method of communication moving forward. PCPs were asked to comment on the most significant benefits and harms of LCS and BP medications and how these benefits and risks compare. PCPs were also asked how worried they were about patients dying from lung cancer without screening and having a myocardial infarction or stroke before starting BP medication. These interviews were audio recorded. Because the physicians were VA employees, they were not eligible to receive compensation for participating.

### Analysis Plan

The study principal investigator and research team analysts had access to the deidentified dataset. Analyses included all participants who completed the medical encounter. One patient missed their survey and interview due to a conflict, whereas 3 medical encounters were incomplete—2 (67%) due to technical issues and 1 (33%) because the patient opted out of recording. These data were reported as missing in the analysis.

The medical encounters, patient interviews, and physician interviews were all audio recorded and transcribed verbatim. An internal transcriptionist transcribed patient interviews, whereas the medical encounters and PCP interviews were transcribed through the Microsoft Teams transcription software. Transcripts were analyzed using a deductive approach, and a preliminary codebook was developed based on the interview guide. Transcripts were coded using Microsoft Excel by a team of trained qualitative analysts who individually coded qualitative data and met weekly to address discrepancies and improve interrater reliability. After discrepancies were addressed, a new Microsoft Excel form was populated, a culmination of both qualitative coders’ work. Following coding, the team applied a rapid matrix analysis framework to identify common themes and patterns among the data related to how patients and physicians viewed the acceptability and feasibility of the approach and the physician’s fidelity to it. This analysis was completed in NVivo (Lumivero). The audio-recorded medical encounters were also used to deduce the length of time of the entire appointment, the length of the initial recommendation, and the length of the entire ZIP conversation. Analysis of the patient and physician survey data was double entered by analysts and included basic descriptive statistics (eg, means, frequencies, and SDs). We analyzed important demographic and clinical characteristics, including age, sex, race, and general health status, to explore variations in responses. The survey used validated tools to assess individual preferences for seeking medical care and how patients perceive SDM. The quantitative results were combined with qualitative themes to offer contextual insights and identify potential differences in the experiences of various subgroups (eg, encouragement vs preference-sensitive zones).

## Results

### Overview

Data collection began in October 2023 and concluded in April 2024. Data analysis concluded in December 2024. Paper publication is expected in Summer 2025.

### Patient and Physician Characteristics

This study was funded in October 2022 by the VA. After obtaining IRB approval for all study procedures and documents, PCP recruitment and enrollment began in July 2023. Patient recruitment then began in October 2023 after recruiting the physicians and engaging them in the education sessions.

A total of 10 PCPs were recruited and enrolled in this study. Half (n=5, 50%) were male, and most (n=8, 80%) reported being either White or Asian individuals. In total, 40% (4/10) of the physicians had completed their medical training more than 20 years before, some (n=3, 30%) were relatively new to the VA (<5 years), and most (n=7, 70%) worked 3 to 4 days a week in the primary care clinic. Complete physician demographics can be found in Table 2.

**Table 2:** Patient and physician demographics.

				Total		LCS^a^		BP^b^ treatment decisions
**Patients**								
	**Sex, n/N (%)**							
		Male	21/23 (91)		4/4 (100)		17/19 (89)	
		Female	1/23 (4)		0 (0)		1/19 (5)	
		Missing	1/23 (4)		0 (0)		1/19 (5)	
	**Race, n/N (%)**							
		African American	1/23 (4)		0 (0)		1/19 (5)	
		White	21/23 (91)		4/4 (100)		17/19 (89)	
		Missing	1/23 (4)		0 (0)		1/19 (5)	
	Age (y), mean (SD)			69 (6.6)		66 (6.5)		70 (6.6)
	**Educational level, n/N (%)**							
		High school	4/23 (17)		0 (0)		4/19 (21)	
		Some college or trade school	10/23 (43)		3/4 (75)		7/19 (37)	
		Associate’s degree	4/23 (17)		0 (0)		4/19 (21)	
		Bachelor’s degree	2/23 (9)		1/4 (25)		1/19 (5)	
		Master’s degree or higher	2/23 (9)		0 (0)		2/19 (11)	
		Missing	1/23 (4)		0 (0)		1/19 (5)	
	**Risk zone, n/N (%)**							
		Preference sensitive	10/23 (43)		0 (0)		10/19 (53)	
		Encouragement	13/23 (57)		4/4 (100)		9/19 (47)	
**Physicians, n/N (%)**								
	**Sex**							
		Male	5/10 (50)		―^c^		―	
		Female	5/10 (50)		―		―	
	**Race**							
		African American	0 (0)		―		―	
		Asian	4/10 (40)		―		―	
		White	4/10 (40)		―		―	
		Missing	2/10 (20)		―		―	
	**Hispanic or Latino**							
		Yes	1/10 (10)		―		―	
		No	9/10 (90)		―		―	
	**Time since completion of clinical training (y)**							
		<5	2/10 (20)		―		―	
		5-10	2/10 (20)		―		―	
		11-19	2/10 (20)		―		―	
		≥20	4/10 (40)		―		―	
	**Duration practicing at the VA^d^ (y)**							
		<5	3/10 (30)		―		―	
		5-10	1/10 (10)		―		―	
		11-19	6/10 (60)		―		―	
		≥20	0 (0)		―		―	
	**Average day per week working in primary care**							
		1-2	3/10 (30)		―		―	
		3-4	7/10 (70)		―		―	
		≥5	0 (0)		―		―	

^a^LCS: lung cancer screening.

^b^BP: blood pressure.

^c^Not applicable.

^d^VA: US Department of Veterans Affairs.

**Figure 2:**
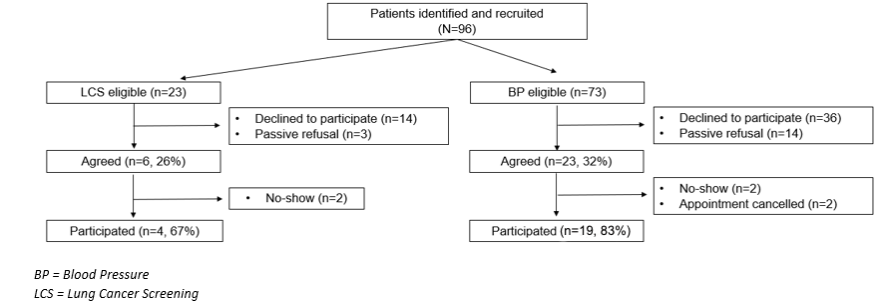
Consolidated Standard of Reporting Trials (CONSORT) diagram.

From the physician panels, 96 patients were identified as eligible for the study: 23 (24%) were eligible for an LCS conversation, and 73 (76%) were eligible for a BP conversation. Within the LCS-eligible cohort, of the 23 patients, 6 (26%) agreed to the study over the phone, 14 (61%) declined to participate, and 3 (13%) were unable to be reached via telephone. Of the 6 patients who agreed, only 4 (67%) consented and participated as 2 (33%) were no-shows to their appointments. Regarding the BP-eligible cohort, of the 73 patients, 23 (32%) agreed over the phone, of whom 19 (83%) were consented and participated (n=2, 9% of the patients were no-shows and n=2, 9% canceled their appointments). Of the 73 eligible BP treatment decision patients, 36 (49%) declined to participate, and 14 (19%) were unable to be reached via telephone. Complete recruitment details can be found in Figure 2.

Of the 23 total participants, most were male (n=21, 91%) and White individuals (n=21, 91%), the mean age was 69 (SD 6.6) years, and more than three-quarters (18/23, 78%) completed at least some college. Within the BP cohort, 53% (10/19) of the participants were in the preference-sensitive zone, and 47% (9/19) were in the encouragement zone. All 4 LCS patients were identified in the encouragement zone. Complete demographic details can be found in Table 2.

Not all physicians were able to see an LCS patient. Of the 10 recruited physicians, 4 (40%) saw 3 total patients (n=2, 67% for BP treatment decisions and n=1, 33% for LCS). The remaining physicians saw either 1 or 2 BP treatment decision patients and no LCS patients. Full details on the patient distribution by PCP can be found in Table 3.

**Table 3:** Patient distribution by physician.

Physician ID	Number of LCS^a^ patients	Number of BP^b^ treatment decision patients	Total
1	1	2	3
2	1	2	3
3	1	2	3
4	1	2	3
5	0	2	2
6	0	2	2
7	0	2	2
8	0	2	2
9	0	2	2
10	0	1	1

^a^LCS: lung cancer screening.

^b^BP: blood pressure.

### Protocol Challenges

One of the main challenges encountered throughout this protocol was recruitment. A specific challenge was the identification of eligible LCS patients, likely due to long-standing LCS at the site and most patients on these physicians’ panels already having started LCS. During each 2-week eligibility pull, there were typically 1 to 3 eligible LCS patients identified, with a high of 6 patients identified in 1 pull. This resulted in a very small pool of recruitable patients, leading to low recruitment numbers and, ultimately, lack of success in recruiting an LCS patient for every recruited physician. In addition, there were several instances in which a recruited patient’s appointment was canceled or rescheduled.

## Discussion

### Principal Findings

This study tested the real-world feasibility of the ZIP approach during primary care visits, aiming to assess whether this novel decision-making approach could help bridge implementation gaps left by more time-consuming and impractical SDM approaches. The results of this pilot study will contribute to the ongoing efforts to integrate a feasible SDM approach into routine primary care encounters, particularly within the time and resource constraints of an appointment. The ZIP approach aims to address the challenges of incorporating SDM into brief primary care visits, where PCPs often have limited time to discuss multiple preventive care options and integrate patient preferences into the decision-making process. While the concept of ZIP appeals to patients, the findings of this study will determine the feasibility and acceptability to both patients and physicians of integrating it into practice.

The mixed methods approach involved medical encounters, postencounter surveys and interviews, and physician feedback sessions. The takeaways found in this pilot can inform the actual time taken to deliver ZIP, patient and physician perceptions of the approach, feasibility of physicians’ delivery of the approach, and any specific approach challenges. Findings will be submitted for publication in a peer-reviewed journal. A lay summary will be mailed to research participants.

### Implications for Practice

The findings of this pilot feasibility study also offer insights that will inform the development of the PCP education process and patient identification and recruitment strategies for larger-scale projects. Through feedback obtained from participating physicians, we can improve the education session to address specific challenges and enhance PCPs’ understanding and confidence in implementing the ZIP approach effectively. Specifically, sending the physician the video before the education session to watch on their own time would help alleviate the time burden and the need to schedule a full hour for an education session. It would also allow the physicians time to process the information within the video and come to the session with more thoughtful questions and comments. In addition, insights gained from patient recruitment and identification strategies, including through electronic health records (EHRs) and targeted patient outreach, can be leveraged to optimize recruitment efforts and assess feasibility for future larger-scale studies, such as engaging the physicians in the patient identification process and adding a second staff member to engage with the patients to reduce scheduling conflicts. By using the results of this pilot to refine PCP education processes and recruitment strategies, larger-scale projects can be better equipped to engage physicians and patients effectively.

### Limitations

It is important to acknowledge the limitations of the paper-based prototype used in this study. While effective for the purposes of this pilot project, the final goal is to integrate the decision aid seamlessly into the EHR. Transitioning to an automated tool within the EHR is important as relying on paper-based decision aids that are patient specific and available before clinic visits is unrealistic without additional research staff support. EHR integration can better support providing personalized information to physicians based on the patients’ data within decision support tools that can better fit into the usual EHR-based clinical workflows of modern clinical practice. Future studies will be needed to complete this integration and study the feasibility of carrying out ZIP guided by the support of EHR-integrated decision aid tools. Although we examined both cancer screening and cardiovascular prevention topics, this study’s focus on LCS and BP treatment decisions may not be generalizable to all other preventive care scenarios. Future research can explore the applicability of the ZIP approach across a broader spectrum of preventive care services to assess its versatility and effectiveness in diverse clinical contexts.

### Future Directions

The results of this study will show the potential of the ZIP approach to enhance patient-centered care delivery in the primary care setting. Specifically, these findings can inform the physician education process, patient eligibility criteria, and participant recruitment strategies within larger-scale research. If this pilot demonstrates the feasibility and acceptability of the ZIP approach, it will lay the groundwork for further exploration and refinement of this approach to improving patient engagement and personalizing decision-making within the time constraints of primary care.

## Appendix

Multimedia Appendix 1 Example lung cancer screening decision aid.

Multimedia Appendix 2 Veteran survey.

Multimedia Appendix 3 Veteran interview guide.

Multimedia Appendix 4 Physician interview guide.

Multimedia Appendix 5 Physician survey.

## Abbreviations

BP: blood pressure
DVR: digital voice recorder
EHR: electronic health record
IRB: institutional review board
LCS: lung cancer screening
PCP: primary care physician
SDM: shared decision-making
VA: United States Department of Veterans Affairs
ZIP: Zeroing in on Individualized, Patient-Centered Decisions

## Data Availability

The deidentified data sets generated or analyzed during this study are available from the corresponding author on reasonable request.
