# Supplementary figures and images for: An Everyday Patient-Centered Discussion Model for Primary Care: Protocol for a Feasibility and Acceptability Study of the Zeroing in on Individualized, Patient-Centered Decisions (ZIP) Approach
Source: JMIR Res Protoc. 2025 Oct 8;14:e64998. doi: 10.2196/64998 (PMC12547340; doi:10.2196/64998)

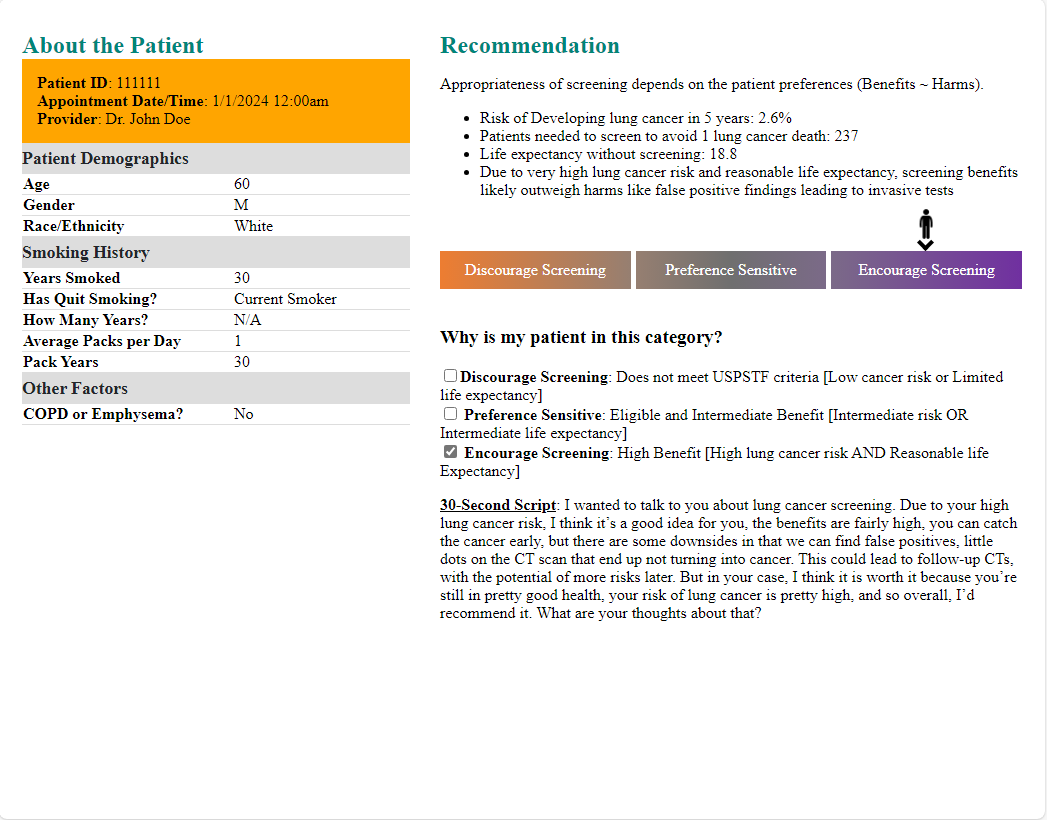
**Multimedia Appendix 1**. Example Lung Cancer Screening Decision Aid

Supplement: Multimedia Appendix 1 [file resprot_v14i1e64998_app1.docx]
