# Supplementary material for: An Everyday Patient-Centered Discussion Model for Primary Care: Protocol for a Feasibility and Acceptability Study of the Zeroing in on Individualized, Patient-Centered Decisions (ZIP) Approach
Source: JMIR Res Protoc. 2025 Oct 8;14:e64998. doi: 10.2196/64998 (PMC12547340; doi:10.2196/64998)
Supplement: Multimedia Appendix 2 [file resprot_v14i1e64998_app2.docx]

**Multimedia Appendix 2**. Veteran Survey

**Veteran Survey**

**Participant ID Number: ____________**

**[Questions include Lung Cancer Screening and starting and stopping Blood Pressure Treatment, the questions will be separated before administering to the Veteran]**

| **Demographics** |
| --- |

**Q1: Please indicate your year of birth below:**

**___________ (example: 1950)**

**Q2: What is your gender?**

□ Male

□ Female

□ Other/Transgender

**Q3: Are you Hispanic or Latino?** (Defined as a person of Cuban, Mexican, Puerto Rican, South or Central American, or other Spanish culture or origin, regardless of race)

□ No

□ Yes

**Q4: What is your race?**

□ American Indian or Alaska Native

□ Asian

□ Black or African American

□ Native Hawaiian or Other Pacific Islander

□ White

□ Other (Please list) ___________________________

**Q5: What is the highest degree or level of education you have completed?**

□ Less than high school

□ High school graduate (diploma or GED)

□ Some college/trade school

□ Associate’s degree

□ Bachelor’s degree

□ Master’s degree or more

**Q6: What is your marital status?**

□ Single (never married)

□ Married

□ Separated

□ Widowed

□ Divorced

| **General Health Information** |
| --- |

**Q7: In general, would you say your health is:**

□ Excellent

□ Very good

□ Good

□ Fair

□ Poor

**Q8. How often do you need to have someone help you when you read instructions, pamphlets, or other written material from your doctor or pharmacy?**

□ Never

□ Rarely

□ Sometimes

□ Often

□ Always

| **Q9: How good are you at working with fractions?** | | | | | | | | | | | |
| --- | --- | --- | --- | --- | --- | --- | --- | --- | --- | --- | --- |
| **1** | **2** | | | **3** | | **4** | | **5** | | | **6** |
| **Not good at all** | |  |  | |  |  |  | |  | **Extremely good** | |

| **Q10: How good are you at figuring out who much a shirt will cost if it is 25% off?** | | | | | | | | | | | |
| --- | --- | --- | --- | --- | --- | --- | --- | --- | --- | --- | --- |
| **1** | **2** | | | **3** | | **4** | | **5** | | | **6** |
| **Not good at all** | |  |  | |  |  |  | |  | **Extremely good** | |

| **Q11: How often do you find numerical information to be useful?** | | | | | | | | | | | |
| --- | --- | --- | --- | --- | --- | --- | --- | --- | --- | --- | --- |
| **1** | **2** | | | **3** | | **4** | | **5** | | | **6** |
| **Never** | |  |  | |  |  |  | |  | **Very Often** | |

| **Satisfaction with and General Preferences for Health Care** |
| --- |

| **Q12: All things considered, on a scale from 1-10, how satisfied are you with your health care in the VA?** | | | | | | | | | |
| --- | --- | --- | --- | --- | --- | --- | --- | --- | --- |
| **1** | **2** | **3** | **4** | **5** | **6** | **7** | **8** | **9** | **10** |
| **Completely**  **Dissatisfied** | |  |  |  |  |  |  | **Completely Satisfied** | |

| **Q13: If I have a medical problem, my preference is to go straight to a doctor and ask his or her opinion.** | | | | | | | | | |
| --- | --- | --- | --- | --- | --- | --- | --- | --- | --- |
| **1** | **2** | **3** | **4** | **5** | **6** | **7** | **8** | **9** | **10** |
| **Strongly Disagree** | |  |  |  |  |  |  | **Strongly**  **Agree** | |

| **Trust** |
| --- |

**Q14: Please indicate how much you disagree or agree with the following statements:**

|  | **Strongly**  **Disagree** | **Somewhat Disagree** | **Neither Agree nor Disagree** | **Somewhat Agree** | **Strongly Agree** |
| --- | --- | --- | --- | --- | --- |
| A. I have complete trust in my doctor to provide the medical care I need. |  |  |  |  |  |
| B. I have complete trust in my local VA Healthcare System to provide the medical care Veterans need. |  |  |  |  |  |
| C. I have complete trust in the national VA Healthcare System to provide the medical care Veterans need. |  |  |  |  |  |

| **Patient-provider Relationship** |
| --- |

**Q15: Please indicate below how much control you like to have when decisions are being made about your medical treatment**

□ I prefer to make the decision about which treatment I receive

□ I prefer to make the final decision about my treatment after seriously considering my doctor’s opinion

□ I prefer that my doctor and I share responsibility for deciding which treatment is best for me

□ I prefer that my doctor makes the final decision about which treatment will be used, but seriously considers my opinion

□ I prefer to leave all decisions regarding treatment to my doctor

**Q16: Please think about your current VA primary care provider when you answer the following four questions:**

|  | **All of the time** | **Most of the time** | **Some of the time** | **Rarely (or Seldom)** | **None of the time** |
| --- | --- | --- | --- | --- | --- |
| A. How often does your doctor offer you choices in your medical care? |  |  |  |  |  |
| B. How often does your doctor discuss the pros and cons of each choice with you? |  |  |  |  |  |
| C. How often does your doctor get you to state which choice or option you prefer? |  |  |  |  |  |
| D. How often does your doctor take your preferences into account when making treatment decisions? |  |  |  |  |  |

| **[Lung Cancer Screening (LCS) /BP Treatment] Decision-making during appointment** |
| --- |

**Q17: Please think about the appointment that was just completed when you answer the following questions:**

|  | **Completely Disagree** | **Strongly Disagree** | **Somewhat Disagree** | **Somewhat Agree** | **Strongly Agree** | **Completely Agree** |
| --- | --- | --- | --- | --- | --- | --- |
| A. My doctor made clear that a decision needs to be made about LCS/BP medication]. |  |  |  |  |  |  |
| B. My doctor wanted to know exactly how I want to be involved in making the decision about [LCS/BP medication]. |  |  |  |  |  |  |
| C. My doctor told me that there are different options for [LCS/BP medication]. |  |  |  |  |  |  |
| D. My doctor precisely explained the advantages and disadvantages of [LCS/ starting or stopping BP medication]. |  |  |  |  |  |  |
| E. My doctor helped me understand all the information |  |  |  |  |  |  |
| F. My doctor asked me which treatment option I prefer. |  |  |  |  |  |  |
| G. My doctor and I thoroughly weighed the different treatment options. |  |  |  |  |  |  |
| H. My doctor and I selected a treatment option together. |  |  |  |  |  |  |
| I. My doctor and I reached an agreement on how to proceed. |  |  |  |  |  |  |

**Q18: Did you healthcare provider give you a recommendation about whether or not to [get a lung cancer screening test/start or stop a blood pressure regulating medication]?**

*For LCS Patient***:**

□ No, my provider did not make a recommendation (If no, please skip following question)

□ Yes, recommended that I NOT be screened

□ Yes, recommended that I be screened

*For Starting BP Medication*:

□ No, my provider did not make a recommendation (If no, please skip following question)

□ Yes, recommended that I NOT start taking a medication

□ Yes, recommended that I start taking a medication

*For Stopping BP Medication*:

□ No, my provider did not make a recommendation (If no, please skip following question)

□ Yes, recommended that I NOT stop taking a medication

□ Yes, recommended that I stop taking a medication

| **Q18a: If you received a recommendation, how strong was the recommendation your provider gave you?** | | | | | | | | | |
| --- | --- | --- | --- | --- | --- | --- | --- | --- | --- |
| **1** | **2** | **3** | **4** | **5** | **6** | **7** | **8** | **9** | **10** |
| **Not at all strong** | |  |  |  |  |  |  | **Very Strong** | |

| **Q18b: If you received a recommendation, did you feel like you could disagree with your provider’s recommendation?** | | | | | | | | | |
| --- | --- | --- | --- | --- | --- | --- | --- | --- | --- |
| **1** | **2** | **3** | **4** | **5** | **6** | **7** | **8** | **9** | **10** |
| **Could definitely NOT disagree** | |  |  |  |  |  |  | **Could definitely disagree** | |
