# Supplementary material for: An Everyday Patient-Centered Discussion Model for Primary Care: Protocol for a Feasibility and Acceptability Study of the Zeroing in on Individualized, Patient-Centered Decisions (ZIP) Approach
Source: JMIR Res Protoc. 2025 Oct 8;14:e64998. doi: 10.2196/64998 (PMC12547340; doi:10.2196/64998)
Supplement: Multimedia Appendix 3 [file resprot_v14i1e64998_app3.docx]

**Multimedia Appendix 3**. Veteran Interview Guide

**Introduction**

Thank you for completing the survey, I just have a few follow-up questions I wanted to discuss with you. This interview should only take about 10-15 minutes. Our goal is just to understand more about your thought process with decision making, perspectives on benefits and harms, and interactions with your provider. As we have mentioned before, all of this will remain completely confidential and will not get back to your provider. Therefore, feel free to answer openly and honestly. But you are free to not answer any questions you would not like to.

As I mentioned, I will be recording this, so we have an accurate record of what you tell us. If you would like me to pause or turn off the recorder at any time, please let me know.

***Turn recorder on***

This is participant # _____

**Everyday SDM Debrief**

1. How long have you been seeing your current provider? Dr. _____?
2. During your appointment did you doctor discuss [LCS/BP treatment] options with you?
   1. If so,
      1. How did the conversation go?
   2. If no – Skip to Decision- Making questions
3. Did you have any questions about [LCS/BP treatment] for your doctor after the conversation?
   1. Do you have any questions that you didn’t ask your doctor that you wish you would have?
4. How strongly did you feel your provider either recommended or didn’t recommend [LCS/BP treatment]?
   1. Tell me more about why you felt that?
5. We have been training some providers in a new communication technique to include more personalization in their recommendations, do you feel the conversation was personalized to you?
   1. Was this reflected in your conversation?
6. Would you like to see this personalized communication approach used more frequently with other preventive care topics?

**Decision Making**

1. I see here on the survey you answered (Q15 about control with medical decisions) Will you please tell me more about how you like to make medical decisions? Why?
   1. Does your control over decision making change based on the health topic or health concern?
2. At this time have you made a decision about [LCS/BP treatment] at this time?
   1. Why did you make this decision?

**Benefits/Harms**

1. What do you think are potential benefits of [LCS/starting BP treatment]?
   1. What do you think is the most important Benefit? Why?
2. What do you think are potential harms of [LCS/starting BP treatment]?
   1. How worried are you about experiencing a harm? Why?
3. In your opinion, how do you think the risks compare to the benefits?
4. For LCS Only: How worried are you about lung cancer? Why?
   1. In your opinion, how helpful do you think early detection through screening is?
5. For BP Only: How worried are you about heart attacks and strokes?
   1. In your opinion, how helpful is adding a BP medication?

**Conclusion**

That is all of the questions I wanted to ask you. Do you have anything else you would like to add? Do you have any questions for me?

***Turn recorder off***

Thank you very much for participating in this survey and interview. We greatly appreciate your time and thoughtful input. Here is your $25 gift card as a thank you.
