# Supplementary material for: An Everyday Patient-Centered Discussion Model for Primary Care: Protocol for a Feasibility and Acceptability Study of the Zeroing in on Individualized, Patient-Centered Decisions (ZIP) Approach
Source: JMIR Res Protoc. 2025 Oct 8;14:e64998. doi: 10.2196/64998 (PMC12547340; doi:10.2196/64998)
Supplement: Multimedia Appendix 4 [file resprot_v14i1e64998_app4.docx]

**Multimedia Appendix 4**. Physician Interview Guide

**Provider Interview Guide**

**Introduction.**

Thank you for your participation in the pilot study. Now that you have been using Everyday SDM within in the clinic for a little bit now, we just wanted to follow-up and see how things are going. The goal of the conversation is to understand more about your thought process with decision making, perspectives on benefits and harms, and interactions with your provider. As we have mentioned before, all of this will remain completely confidential. Therefore, feel free to answer openly and honestly. But also you don’t need to answer anything you don’t want to.

As I mentioned, I will be recording this, so we have an accurate record of what you tell us. If you would like me to pause or turn off the recorder at any time, please let me know.

***Turn recorder on***

This is participant #______

**Everyday SDM Approach Debrief**

1. First, please start by telling me how using Everyday SDM in the clinic went?
   1. What was your process?
   2. What was easy?
   3. What was difficult?
   4. What barriers did you run into?
2. How strictly did you stick to the scripts given? Why?
   1. What about the scripts worked?
   2. What didn’t work?
3. With the encourage/preference sensitive zone for the patient. How helpful did you find that? Does it give you a different perspective on benefit for individual patients?
4. How long did it take to complete the Everyday SDM approach?
5. How effective is this approach at accomplishing shared decision making?
   1. Was it missing anything?
6. In your opinion, how receptive were your patients to the conversation?

I thought they were receptive.

- 1. Did you notice any different in their reactions towards either LCS or BP treatment?

1. Outside of the [1-3] patients we gave you information about, did you use this approach with any other patients?
   1. If so, how did it go?
   2. How did you determine the recommendation group?
2. Moving forward, do you see yourself continuing to incorporate this type of communication strategy into your practice?
3. Would you like to see this personalized communication approach used more frequently with other preventive care topics?

**Benefits/Harms of LCS**

I now want to ask you first about some of your perceptions on the benefits and harms of lung cancer screening specially:

1. What do you think is the most important or significant benefit of lung cancer screening? Why?
2. How worried are you about your patients dying from lung cancer without screening? Why?
3. What do you think is the most important or significant harm of lung cancer screening? Why?
4. What is your perception of the balance of risks and benefits for lung cancer screening?

**Benefits/Harms of BP Treatment**

Next I want to ask about some of your perceptions on the benefits and harms of Blood pressure treatment:

1. What do you think is the most important or significant benefit of starting Blood pressure medications? Why?
2. How worried are you about your patients having a heart attack or stroke before starting Blood pressure treatment? Why?
3. What do you think is the most important or significant harm Blood Pressure Treatment Medication? Why?
4. What is your perception of the balance of risks and benefits for Blood pressure treatment?

**Wrap-up**

That is all of the questions I wanted to ask you. Do you have anything else you would like to add? Do you have any questions for me?

Thank you very much for participating in this pilot project and this final interview. We greatly appreciate all of your time and thoughtful input and consideration.
