# Supplementary material for: An Everyday Patient-Centered Discussion Model for Primary Care: Protocol for a Feasibility and Acceptability Study of the Zeroing in on Individualized, Patient-Centered Decisions (ZIP) Approach
Source: JMIR Res Protoc. 2025 Oct 8;14:e64998. doi: 10.2196/64998 (PMC12547340; doi:10.2196/64998)
Supplement: Multimedia Appendix 5 [file resprot_v14i1e64998_app5.docx]

**Multimedia Appendix 5**. Physician Survey

**SWIFT Provider Survey**

**Participant ID Number: ____________**

| **Clinical Experience and Other Demographics** |
| --- |

**Q1: What is your gender?**

□ Male

□ Female

□ Other/Transgender

**Q2: Are you Hispanic or Latino?** (Defined as a person of Cuban, Mexican, Puerto Rican, South or Central American, or other Spanish culture or origin, regardless of race)

□ No

□ Yes

**Q3: What is your race?**

□ American Indian or Alaska Native

□ Asian

□ Black or African American

□ Native Hawaiian or Other Pacific Islander

□ White

□ Other (Please list) ___________________________

**Q4: How long ago did you complete clinical training?**

□ <5 years

□ 5-10 years

□ 11-19 years

□ 20+ years

**Q5: How long have you been practicing at the VA?**

□ <5 years

□ 5-10 years

□ 11-19 years

□ 20+ years

**Q: In an average week, approximately how many day or half days do you provide primary care in VA?**

____________ Half days OR ____________ Days
